# Impact of Variants, Epidemiological Trends, and Comorbidities on Hospitalization Rates of Unvaccinated Children in Brazil: A Retrospective Study (2020–2022)

**DOI:** 10.1111/irv.70011

**Published:** 2024-12-16

**Authors:** Danielle Dias Conte, Raí André Silva Watanabe, Ana Paula Cunha Chaves, Felipe Alberto‐Lei, Ana Helena Sita Perosa, Gabriela Barbosa, Nancy Bellei

**Affiliations:** ^1^ Infectious Diseases Division Universidade Federal de São Paulo, UNIFESP São Paulo Brazil

**Keywords:** children, coinfection, comorbidities, COVID‐19, vaccine

## Abstract

This retrospective study aimed to investigate the impact of the emergence of new variants and the epidemiological scenario on hospitalization rates of unvaccinated children (0–12 years) in Brazil. The study included 1614 children admitted to a hospital between March 2020 and December 2022 but 101 (6.3%) of them testing positive for COVID‐19 via RT‐PCR. The frequency of COVID‐19 cases increased from 7.5% in 2020 to 9.3% in 2022 with the emergence of the Omicron variant. Children over 5 years old with comorbidities accounted for most cases (69% [70/101]). Sickle cell anemia was the most frequent comorbidity (20%), and influenza‐like illness (36% [36/101]) and decompensation of underlying disease (33% [33/101]) were the main reasons for hospitalization. Coinfection was detected in 11% of cases, with respiratory syncytial virus (RSV) being the most common viral pathogen (71%). Hospital readmission occurred in 26% of cases, with a higher frequency in children over 5 years old. The death rate was 1.9%, with comorbidities such as cystic fibrosis and congenital heart disease as risk factors. These findings emphasize the need to prioritize vaccination with monovalent Omicron XBB for high‐risk groups, including children over 5 years old with comorbidities, to mitigate the impact of new variants and reduce severe disease outcomes.

## 1 Introduction

Since the beginning of the pandemic, epidemiologic data have shown that hospitalization of children and adolescents associated with the severe acute respiratory syndrome (SARS) due to COVID‐19 did not significantly impacted this population. They differ from the elderly and adult population with comorbidities, which are specially more susceptible to severe disease progression and complications [1].

In children, several factors contributed to milder infection, which reduces the number of the significant receptor angiotensin‐converting enzyme 2 (ACE2) on the cell surface, resulting in less availability of the receptor, and the presence of other respiratory viruses competing simultaneously and limiting replication of different viruses. In neonates and infants, maternal antibodies provide protective immunity against disease [2, 3].

In Brazil, by the end of this study, the incidence of SARS associated with COVID‐19 totaled 21.278 cases and 875 deaths in the age group between 0 and 19 years by epidemiological week 52 of 2022 [1]. In this context, it can also be observed that children were not initially affected by SARS‐CoV‐2 as much as in the 2009 pandemic caused by H1N1 [3].

A recent study reported a change in the current pandemic scenario, showing a significant increase in the proportion of pediatric cases after the emergence of the Omicron variant, rising from > 2% at the beginning of the pandemic to 25% by March 2022 [4].

It should be noted that the government took several preventive measures to reduce transmission rates in the early stages of the pandemic and after the initial outbreak in Brazil. This dramatically impacted the child population, as schools were closed and classes moved to remote locations, as well as the introduction of social distancing (lockdown), the use of masks, increased availability of COVID‐19 testing and the establishment of field hospitals.

However, after 2 years of the pandemic, with the return of social activities, the emergence of new variants, mass vaccination of the adult population, and the characterization of a new variant of concern called Omicron in late 2021, there has been an increase in confirmed cases of hospitalized children associated with this new variant and its subtype.

## 2 Methodology

In this retrospective study, 1614 children aged up to 12 years admitted to the Hospital Sao Paulo (HSP) were tested for COVID‐19 by the real‐time polymerase chain reaction (PCR) method from March 2020 to December 2022. Nasal swab samples were collected from patients upon admission. All specimens were stored in 2‐mL sterile Ringer's lactate solution and transported to the Virology Laboratory at the Federal University of São Paulo for testing. RNA from samples was purified using the Quick‐RNA Viral Kit R1035 (Zymo Research, Irvine, CA, USA) according to the manufacturer's instructions. The purified RNA was stored at −80°C. Viral detection was performed using the extracted samples were immediately submitted to RT‐qPCR to detect SARS‐CoV‐2 using the GeneFinder (GeneFinder COVID‐19 Plus Real Amp kit). A positive result was determined with a cycle threshold (Ct) < 40 according to the manufacturer's instructions. Coinfection with other respiratory viruses during this period was also detected by real‐time PCR for human metapneumovirus (hMPV), human respiratory syncytial virus (hRSV), influenza A (FLU‐A) and B (FLU‐B), adenovirus (HAdV), and human rhinovirus (HRV), standardized in our laboratory since the H1N1 pandemic in 2009 [5, 6, 7, 8].

The criteria used to select the sample population were strictly based on age and the requirement of hospitalization for at least 1 day. Data regarding the clinical status of the patients were obtained through their medical records, which included all relevant clinical information and the progression of their condition from initial consultation to final outcomes. We identified three variables to guide our research investigation: the reason for hospitalization, the patient's previous health conditions, and findings during hospitalization. The primary reasons for hospitalization included the presentation of influenza‐like illness (ILI), decompensation of chronic medical conditions, and any other causes identified during clinical assessment.

Length of hospital stay, intensive care unit (ICU) admission, and death were considered for severe outcome analysis. We also investigated viral load (VL) (Ct value), hospital readmission within 6 months, and the SARS‐CoV‐2 variant waves. We categorized the patient's age as follows: less than 1 year, 1–2 years, 2–5 years, and more than 5 years.

The statistical analysis employed various methods to comprehensively examine the dataset. In addition to the simple logistic regression models utilized for both categorical and numerical variables, the analysis incorporated linear regression techniques to explore linear relationships between variables. Pearson's chi‐square test was also employed specifically for numerical variables to assess associations and dependencies within the dataset.

In more complex analyses, such as logistic regression, OR was calculated via statistical software (Jamovi) from the regression coefficients. These data are presented by a binary dependent variable (outcome) and one or more independent variables (predictors). The Pearson correlation was used to compare the length of hospital stay and age. For cases of ICU admission in relation to age, we used binomial logistic regression.

These diverse statistical approaches enabled a thorough investigation of the data, allowing for a robust understanding of the factors influencing the outcomes under study.

The data were organized using Microsoft Excel 365 software spreadsheets for the year 2023. Statistical analysis was performed using the Jamovi software (Version 2.3.13) and a significance level (α) of 0.05, with a confidence interval of 95%. The chart was created using Microsoft Excel 365 software.

All variables were characterized according to their type. Numerical variables were described using the mean (Me) and standard deviation (SD), as well as the median (Md) and interquartile range (IQR). Categorical variables were presented in terms of absolute frequencies and proportions. To analyze the relationship between variables, we employed simple logistic regression models (for age in years) and binomial logistic regression (for age group and population in years).

## 3 Results

The study reports the results of a COVID‐19 test in 1614 hospitalized children, age ranged from 0 to 12 years, Me = 3.97; Md = 3; SD +/−3.97; and IQR (1–8) years with a confirmation rate of 6.3% (101/1614). Male accounted for 59% of infection cases (Table 1).

**TABLE 1:** Characteristics of the study population.

Characteristic	Overall (%)	SARS‐CoV‐2 test result		Odds ratio (CI)	*p*
		Negative (%)	Positive (%)		
	*n* = 1.614	*n* = 1.513 (93.7)	*n* = 101 (6.3)		
Sex					
Male	892 (55.2)	832 (54.9)	60 (59.4)		
Age (years)	Median 3 (IQR = 1–8)	Median 3 (IQR = 1–8)	Median 6 (IQR 1–10)	1.10 (1.00–1.10)	0.044^a^
Age group (years)					
< 1 years	378 (23.4)	352 (93.1)	26 (6.8)	2.54 (1.20–5.35)	0.014
1–2 years	354 (21.9)	344 (97.1)	10 (2.8)	Ref^b^	Ref^b^
2–5 years	286 (17.7)	267 (93.3)	19 (6.6)	2.44 (1.12–5.35)	0.025
> 5 years	596 (36.9)	550 (92.2)	46 (7.7)	2.88 (1.43–5.78)	0.003
Distribution per years					
2020	**307 (19.0)**	**284 (92.5)**	**23 (7.5)**	2.2 (1.24–3.90)	0.007
< 1 years	65	61	4 (6.5)		
1–2 years	75	73	2 (2.66)		
2–5 years	59	54	5 (9.47)		
> 5 years	108	96	12 (11.11)		
2021	**762 (47.2)**	**735 (96.4)**	**27 (3.5)**	Ref	—
< 1 years	213	208	5 (2.34)		
1–2 years	160	159	1 (0.62)		
2–5 years	118	113	5 (4.23)		
> 5 years	271	255	16 (5.9)		
2022	**545 (33.7)**	**494 (90.6)**	**51 (9.3)**	2.81 (1.73–4.54)	< 001
< 1 years	100	83	17 (17)		
1–2 years	109	102	7 (6.42)		
2–5 years	119	110	9 (7.56)		
> 5 years	217	199	18 (8.29)		

*Note:* Estimated odds ratio and *p* values using simple logistic regression model (age years)^a^ and binominal logistic regression (age group and population of years)^b^.

Abbreviation: Ref, reference.

Group of 1–2 years of age had the lowest frequency of cases of COVID‐19 infection among age groups (Table 1). Increase age did not showed a significant difference (*p* 0.044; OR = 1.10; CI 95% [1.00–1.10]).

There was no statistically significant association between COVID‐19 infection and the following causes, comorbidities, hospitalization, ILI syndrome, ICU, and high VL (CT value) (Table 2).

**TABLE 2:** Characteristics of positive cases.

Overall (%)					Total (%)	Odds ratio (CI)	*p*
	< 1 years	1–2 years	2–5 years	> 5 years			
Comorbidities							
	12/70 (17)	7/70 (10)	13/70 (19)	38/70(54)	70/10 1(69)	0.86 (0.77–0.97)	0.055
Intensive care unit							
	6/17 (35)	2/17 (12)	2/17 (12)	7/17 (41)	17/101 (17)	0.98 (0.86–1.11)	0.760
Hospital admission							
Influenza like illness	13/36 (36)	3/36 (8)	2/36 (6)	18/36 (50)	36/101 (36)	1.01 (0.92–1.11)	0.727
Decompensation of the underlying disease	6/33 (18)	4/33 (12)	8/33 (24)	15/33 (46)	33/101 (33)	1.02 (0.91–1.15)	0.655
Other causes	7/32 (22)	3/32 (9)	9/32 (28)	13/32 (41)	32/101 (32)	0.99 (0.88–1.12)	0.949
Hospitalized readmission							
	3/26 (11.5)	6/26 (23.1)	5/26 (19.2)	12/26 (46.2)^a^	26/101 (26)	4.25 (1.02–17.69)	0.005^a^
Coinfection cases							
	7/11 (64)	1/11 (9)	1/11 (9)	2/11 (18)	11/101 (11)	—	

*Note:* The number among relatives is the percentage (%).

^a^ Estimated odds ratio and *p* values using simple logistic regression model (age years) and binominal logistic regression (age group and population of years).

The main reason for hospitalization was ILI with 36% rate (36/101), decompensation of the underlying disease with 33% (33/101), and other causes not related to risk factors for COVID‐19 with 32% (32/101) (Table 2). Sixty‐nine percent (70/101) of patients had underlying comorbidities as a risk factor for COVID‐19, and the most common comorbidities were sickling cell anemia (20%), congenital heart disease (CHD) (12%), asthma (9.7%), and diabetes (9.7%).

The hospitalization length of stay ranged from 1 to 240 days (Me = 13.7; SD +/−31.85; Md = 6; IQR (4–9) days). Seventeen percent (17/101) of the patients required ICU (Me = 4.9; SD+/−4.27; Md = 4; IQR [3–10]), and the length of stay in the ICU ranged from 1 to 40 days (Me = 9.14; SD +/−10.84; IQR [3–10] days). Among these cases, 24% (4/17) of the patients were admitted due to decompensation of the underlying disease, but the highest reason rate was ILI, representing 47% (8/17) of ICU patients. There was no correlation between the length of hospital stay and age, nor was there any association between ICU admission and age.

The patient's VL was determined using a proxy the CT value, which is inversely proportional to the VLs. This means that the lower the CT value, the higher the VL. The mean CT value of the patients' samples was 27.57 with a standard deviation of +/−6.71. The median CT value was 29, and the IQR was 22 to 34.

The hospitalization due to ILI had a median CT value of 25 and an IQR of 20.5–31, with a mean of 25.17 and a standard deviation (SD) of +/−6.42. Exacerbation of chronic diseases hospitalization cases obtained median of 33 and an IQR of 23–35, with a mean of 29.27 and an SD of +/−6.72. For other inpatients samples (other reasons), the CT value had a median of 30.5 and an IQR of 24–33.5, with a mean of 28 and an SD of +/−6.21. There was a not statistically significant association between ILI and CT value (*p* = 0.028; OR = 1.072; CI 95% [1.00–1.14]).

Death occurred in 2 cases (1.9%), both presenting comorbidities. One case occurred in 2021, the patient had cystic fibrosis; hospitalization was due to the decompensation of the underlying disease and remained in the ICU until death. The other case occurred in 2022; the patient with CHD was admitted for decompensation of the underlying disease.

Hospital readmission occurred in 26% (26/101) of the cases, with higher frequency in children over 5 years (46%). This group showed a statistically significant association with readmission (*p* = 0.005; OR 4.25; CI95% [1.02–17.69]).

The frequency of COVID‐19 cases among the years analyzed 2020, 2021, and 2022 was 7.5% (23/307), 3.5% (27/762), and 9.3% (51/545), respectively.

We observed a statistically significant contrast between the infection rates observed in 2020 and 2022 compared to 2021. Children admitted to hospital in 2020 were 2.2 times more likely to contract COVID‐19 than those admitted in 2021, *p* = 0.007; OR 2.2; CI95% (1.24–3.50), whereas children hospitalized in 2022 had 2.8 times higher odds of infection compared to their counterparts in 2021 *p* < 001; OR 2.8; CI95% (1.73–4.54).

The frequency of coinfection during this period was 11% (11/101). RSV occurred in 64% (7/11), four cases in children up to 1 year, one case in 2–5 years, and two cases over 5 years. HRV was detected in 27% (3/11), and the detection of Flu‐A coinfected a child of 2 years old.

**FIGURE 1:**
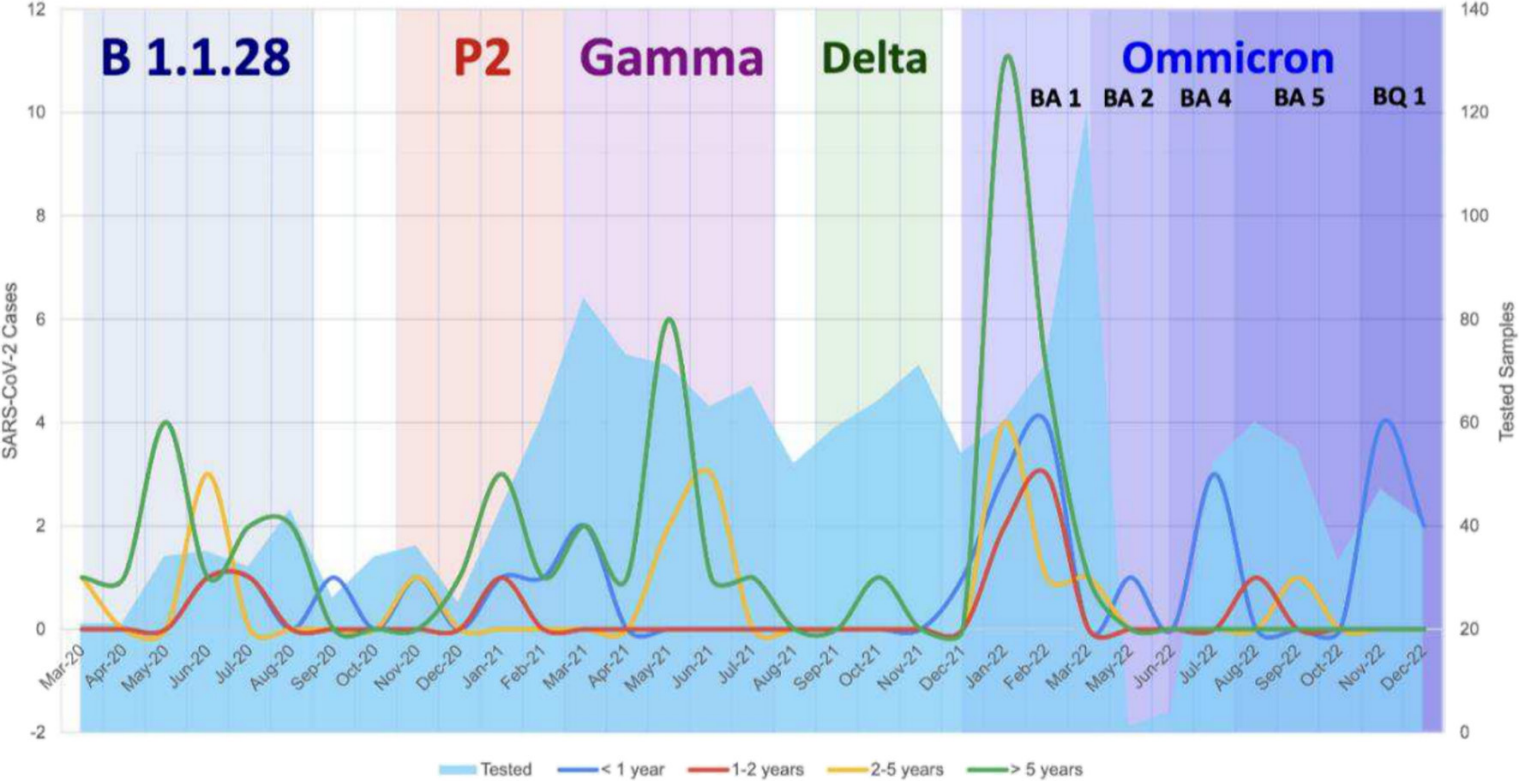
Graph indicating the number of positive cases per age group and their monthly distribution since the onset of the COVID‐19 pandemic.

The following graph (Figure 1) illustrates positive cases across different age groups and their monthly distribution since the onset of the pandemic. The peaks of cases in children younger than 5 years coincided with the emergence and spread of new variants in Brazil, whereas in children older than 5 years, there is continuous detection throughout all these periods.

## 4 Discussion

In this cohort retrospective study, the characteristics of the population of children up to 12 years of age hospitalized and diagnosed with COVID‐19 at HSP were evaluated. It is noteworthy that patients treated and hospitalized at HSP present with complex conditions. In this analysis, a portion of the population was eligible for vaccination, but none had completed vaccination during hospitalization. Vaccination for children between 5 and 11 years of age was not approved until the beginning of 2022, and for children between 6 months and 4 years of age, it was not approved until the end of last year. Since the onset of this pandemic, children have not been as susceptible to the virus as adults, as seen in the last pandemic in 2009 caused by the H1N1 influenza virus, which severely affected children at the time [9].

However, this scenario has changed, possibly due to the high vaccination coverage in the adult and elderly population, immunization of children older than 12 years until the end of 2021, the lifting of quarantine measures, and the emergence of new variants. In this new context, our study showed that children up to 11 years of age became more susceptible to the new circulating Omicron variant. A recent study reported a shift in the current pandemic scenario, indicating a significant increase in the proportion of pediatric cases after the emergence of the Omicron variant, rising from > 2% at the beginning of the pandemic to 25% by March 2022 [4].

As observed in our study, most patients were hospitalized for other causes or exacerbation of underlying diseases with an acute respiratory infection.

Ungar et al. investigated the relationship between COVID‐19 and comorbidities in children and admission to ICU, 2020–2021, but Omicron variants were not recorded during their study period. Hospitalizations were more frequent among children under 5 years old, contrary to what was observed in our study, where the highest frequency of COVID‐19 cases was observed in children over 5 years old. Their study differed from ours in the frequency of comorbidities (29.7% vs. 69%) among older children [10].

Other studies reported that although most cases are mild, children with underlying conditions are at higher risk of developing severe symptoms of the disease [4]. In Brazil, one study reported that approximately 40% of the pediatric population had at least one chronic condition, 14.6% associated with neurological conditions, and 14.2% of children diagnosed with two or more chronic conditions as a risk factor for COVID‐19 [9].

International data have presented a panorama similar to the results found in our study. In South American countries, cases of COVID‐19 have been observed in children. A study in Ecuador, led by Ortiz‐Prado, examined the epidemiology of the disease in children (0–19 years) at the beginning of the pandemic. Adolescents aged 15–19 years were more susceptible to infection, children under 1 year of age had the highest mortality rate, a higher risk of mortality compared to older children and adolescents [11].

The data from various studies provide insights into COVID‐19 cases among children. In Colombia [12], Bolanos‐Almeida et al. found that 9.2% were in children under 18, with most cases being mild or asymptomatic and few requiring hospitalization or ICU admission. In Spain, Garcia‐Vera et al. [13] observed that 50.3% were asymptomatic and symptomatic cases more prevalent in younger children. Hospitalization rates were low, with only two cases requiring ICU. The Children's Hospital of Philadelphia (CHOP) study tested 7256 children, finding a 5.8% positivity rate, highest among older age groups. Hospitalization rates were higher in younger children, with two recorded deaths. Overall, the data suggest that older children, particularly those over 5 years old, have the highest prevalence of infection, with each additional year of age increasing the likelihood of infection by approximately 10% [14].

Our research, consistent with previous studies, highlights that the age group most affected by SARS‐CoV‐2 infection is children over 5 years old. This study offers valuable insights into the risk factors for severe COVID‐19 in children and underscores the importance of ongoing monitoring for long‐term outcomes in this population.

Sickle cell anemia was found to be the most prevalent comorbidity among children in our study, affecting 20% of cases, followed by CHD at 12%. These findings are supported by other studies.

A review conducted by Rocha et al. explored the relationship between sickle cell anemia and COVID‐19. The study revealed that patients with sickle cell anemia are at high risk of contracting SARS‐CoV‐2 due to their compromised immunity, leading to severe complications and multiorgan failure, especially when combined with COVID‐19 [15].

Mucalo et al. presented data from 1045 confirmed COVID‐19 cases over 2 years, with 590 (56.5%) being children with the HbSS genotype for sickle cell anemia. The majority experienced mild symptoms, and 383% were hospitalized, and 14.6% were transferred to the ICU [16].

In the limited literature available, there are indications that patients with CHD may face an elevated risk of cardiovascular complications and ICU admission due to COVID‐19. Sabatino et al. found that most cardiovascular complications occurred in CHD patients with confirmed COVID‐19 cases, including heart failure (55%), arrhythmias (22%), and stroke (22%) [17]. Similarly, Simpson et al. reported a 71% ICU admission rate among patients aged 3 months to 19 years with both CHD and COVID‐19 [18].

In our study, we observed a low proportion of coinfection (11%), which contrasts with other reports. Another Brazilian study conducted by Bain et al. found a 20% rate of coinfection, mainly involving Influenza B and fewer RSV cases, likely due to a large inclusion of older children and adolescents [19].

This study provides valuable insights into the risk factors for severe COVID‐19 in children and highlights the importance of continuous monitoring of long‐term outcomes in this population. Notably, 69% of children had comorbidities, with 22.5% having more than one, and 25.71% were readmitted. A meta‐analysis found that the risk of ICU admission increased with the number of comorbidities, and vaccination was effective in reducing hospitalization risk. [20]. Many cases may be associated with long‐term COVID, with recent studies suggesting factors such as age, the presence of comorbidities, severity of acute illness, and obesity contributing to prolonged development of the disease [20, 21]. This information can inform public health policies and recommendations for preventing and treating COVID‐19 in children, emphasizing the importance of prioritizing vaccination and providing periodic follow‐up for cases associated with long‐term COVID‐19.

With the natural evolution of SARS‐CoV‐2, driven by evolutionary pressure, the emergence of new variants and subtypes, and the increasing number of cases among children, as seen after the Omicron variant, it is challenging to predict the long‐term impact of this disease on children and its effect on the seasonality of other respiratory viruses and epidemiological control in this population.

In conclusion, following the evolution of SARS‐CoV‐2, children were impacted as expected, similar to other respiratory viruses. Our study demonstrated that most hospitalized cases had comorbidities, especially patients with sickle cell anemia, who were frequently readmitted. This suggests that these children should be prioritized for a robust immunization program. The Omicron variant led to the highest rate of hospitalization, indicating the need for the best formulation for children. Therefore, the new monovalent vaccine with Omicron XBB may be the optimal option, particularly for those with comorbidities.

In this context, several research, as well as ours, helps in targeted interventions in order to effectively anticipate, respond, and recover from public health emergencies through scientific knowledge combined with government planning and interventions with communities, at the local, regional, national, and international levels.

This study had several limitations. Firstly, it did not adhere to a controlled research design but relied on spontaneous demand. Secondly, the sample size fluctuated throughout the study period, depending on the increasing number of cases and circulating variants, which could potentially influence the outcomes.

Additionally, due to the lack of a clear definition of long COVID cases, it was challenging to associate them with hospital readmission cases. This ambiguity also made it difficult to determine the existence of persistent symptoms and the cause of decompensation of the underlying disease in children and adolescents with SARS‐CoV‐2 infection who presented post‐COVID‐19.

## Author Contributions

**Danielle Dias Conte:** conceptualization, data curation, formal analysis, investigation, methodology, visualization, writing – original draft. **Raí André Silva Watanabe:** formal analysis, writing – review and editing. **Ana Paula Cunha Chaves:** formal analysis, writing – review and editing. **Felipe Alberto‐Lei:** visualization, writing – review and editing. **Ana Helena Sita Perosa:** investigation, Validation. **Gabriela Barbosa:** writing – review and editing. **Nancy Bellei:** conceptualization, methodology, resources, supervision, writing – review and editing.

## Ethics Statement

The study was conducted in accordance with the Declaration of Helsinki and approved by the Research Ethics Committee from Universidade Federal de São Paulo (UNIFESP)/HSP (process number CEP 29407720.4.0000.5505).

## Conflicts of Interest

The authors declare no conflicts of interest.

## Data Availability

The data that support the findings of this study are available on request from the corresponding author. The data are not publicly available due to privacy or ethical restrictions.
