# Correction to “Impact of Variants, Epidemiological Trends, and Comorbidities on Hospitalization Rates of Unvaccinated Children in Brazil: A Retrospective Study (2020–2022)”

**DOI:** 10.1111/irv.70084

**Published:** 2025-02-25

Conte, D.D. , Watanabe, R.A.S. , Chaves, A.P.C. , Alberto‐Lei, F. , Perosa, A.H.S. , Barbosa, G. and Bellei, N. (2024), Impact of Variants, Epidemiological Trends, and Comorbidities on Hospitalization Rates of Unvaccinated Children in Brazil: A Retrospective Study (2020–2022). Influenza Other Respi Viruses, 18: e70011, 10.1111/irv.70011.PMC1164995439686624

In the article, the funding information was incomplete.

The funding details read:

This study was supported by FINEP/UFMG/REITORIA/PRPQ (29334)

The funding details should read:

This study was supported by FINEP/UFMG/EDITORIAL/PRPQ (29334) and São Paulo State Research Support Foundation (2023/07391‐7).

The online version of the article has been corrected.

We apologize for this error.

